# Supplementary material for: A Critical View of the Application of the APEX Software (Aqueous Photochemistry of Environmentally-Occurring Xenobiotics) to Predict Photoreaction Kinetics in Surface Freshwaters
Source: Molecules. 2019 Dec 18;25(1):9. doi: 10.3390/molecules25010009 (PMC7017383; doi:10.3390/molecules25010009)
Supplement: Supplementary file 1 [file molecules-25-00009-s001.zip › 3.2.4_gcc-4.4.0.zip!/3.2.4_gcc-4.4.0/3.2.4_gcc-4.4.0/doc/octave/html/interpreter/Installation.html]

Installation - Untitled


Next: Emacs Octave Support,
Previous: Trouble,
Up: Top


---

## Appendix F Installing Octave

Here is the procedure for installing Octave from scratch on a Unix
system.

- Run the shell script configure. This will determine the features
  your system has (or doesn't have) and create a file named
  Makefile from each of the files named Makefile.in.

  Here is a summary of the configure options that are most frequently used
  when building Octave:

  `--prefix=`prefix: Install Octave in subdirectories below prefix. The default value of prefix is /usr/local. `--srcdir=`dir: Look for Octave sources in the directory dir. `--enable-bounds-check`: Enable bounds checking for indexing operators in the internal array classes. This option is primarily used for debugging Octave. Building Octave with this option has a negative impact on performance and is not recommended for general use. `--enable-64`: This is an **experimental** option to enable Octave to use 64-bit integers for array dimensions and indexing on 64-bit platforms. You probably don't want to use this option unless you know what you are doing. If you use `--enable-64`, you must ensure that your Fortran compiler generates code with 8 byte signed `INTEGER` values, and that your blas and lapack libraries are compiled to use 8 byte signed integers for array dimensions and indexing. `--enable-shared`: Create shared libraries (this is the default). If you are planning to use the dynamic loading features, you will probably want to use this option. It will make your .oct files much smaller and on some systems it may be necessary to build shared libraries in order to use dynamically linked functions. You may also want to build a shared version of `libstdc++`, if your system doesn't already have one. `--enable-dl`: Use `dlopen` and friends to make Octave capable of dynamically linking externally compiled functions (this is the default if `--enable-shared` is specified). This option only works on systems that actually have these functions. If you plan on using this feature, you should probably also use `--enable-shared` to reduce the size of your .oct files. `--without-blas`: Compile and use the generic blas and lapack versions included with Octave. By default, configure first looks for blas and lapack matrix libraries on your system, including optimized blas implementations such as the free ATLAS 3.0, as well as vendor-tuned libraries. (The use of an optimized blas will generally result in several-times faster matrix operations.) Only use this option if your system has blas/lapack libraries that cause problems for some reason. You can also use `--with-blas=lib` to specify a particular blas library that configure doesn't check for automatically. `--without-ccolamd`: Don't use CCOLAMD, disable some sparse matrix functionality. `--without-colamd`: Don't use COLAMD, disable some sparse matrix functionality. `--without-curl`: Don't use the cURL, disable the `urlread` and `urlwrite` functions. `--without-cxsparse`: Don't use CXSPARSE, disable some sparse matrix functionality. `--without-umfpack`: Don't use UMFPACK, disable some sparse matrix functionality. `--without-fftw`: Use the included fftpack library instead of the fftw library. `--without-glpk`: Don't use the GLPK library for linear programming. `--without-hdf5`: Don't use the HDF5 library for reading and writing HDF5 files. `--without-zlib`: Don't use the zlib library, disable data file compression and support for recent MAT file formats. `--without-lapack`: Compile and use the generic blas and lapack versions included with Octave. By default, configure first looks for blas and lapack matrix libraries on your system, including optimized blas implementations such as the free ATLAS 3.0, as well as vendor-tuned libraries. (The use of an optimized blas will generally result in several-times faster matrix operations.) Only use this option if your system has blas/lapack libraries that cause problems for some reason. You can also use `--with-blas=lib` to specify a particular blas library that configure doesn't check for automatically. `--without-framework-carbon`: Don't use framework Carbon headers, libraries and specific source code for compilation even if the configure test succeeds (the default value is `--with-framework-carbon`). This is a platform specific configure option for Mac systems. `--without-framework-opengl`: Don't use framework OpenGL headers, libraries and specific source code for compilation even if the configure test succeeds. If this option is given then OpenGL headers and libraries in standard system locations are tested (the default value is `--with-framework-opengl`). This is a platform specific configure option for Mac systems. `--help`: Print a summary of the options recognized by the configure script.

  See the file INSTALL for more general information about the
  command line options used by configure. That file also contains
  instructions for compiling in a directory other than where the source
  is located.- Run make.

    You will need a recent version of GNU Make. Modifying Octave's
    makefiles to work with other make programs is probably not worth
    your time. We recommend you get and compile GNU Make instead.

    For plotting, you will need to have gnuplot installed on your system.
    Gnuplot is a command-driven interactive function plotting program.
    Gnuplot is copyrighted, but freely distributable. The `gnu' in gnuplot
    is a coincidence—it is not related to the GNU project or the FSF in
    any but the most peripheral sense.

    To compile Octave, you will need a recent version of GNU Make. You will
    also need a recent version of `g++` or other ANSI C++ compiler. You
    will also need a Fortran 77 compiler or `f2c`. If you use
    `f2c`, you will need a script like `fort77` that works like a
    normal Fortran compiler by combining `f2c` with your C compiler in
    a single script.

    If you plan to modify the parser you will also need GNU `bison` and
    `flex`. If you modify the documentation, you will need GNU
    Texinfo, along with the patch for the `makeinfo` program that is
    distributed with Octave.

    GNU Make, `gcc`, and `libstdc++`, `gnuplot`,
    `bison`, `flex`, and Texinfo are all available from many
    anonymous ftp archives. The primary site is ftp.gnu.org, but it
    is often very busy. A list of sites that mirror the software on
    ftp.gnu.org is available by anonymous ftp from
    ftp://ftp.gnu.org/pub/gnu/GNUinfo/FTP.

    You will need about 1 gigabyte of disk storage to work with when
    building Octave from source (considerably less if you don't compile with
    debugging symbols). To do that, use the command

    ```
              make CFLAGS=-O CXXFLAGS=-O LDFLAGS=
    ```

    instead of just make.- If you encounter errors while compiling Octave, first check the list of
      known problems below to see if there is a workaround or solution for
      your problem. If not,
      see Trouble,
      for information about how to report bugs.- Once you have successfully compiled Octave, run make install.

        This will install a copy of Octave, its libraries, and its documentation
        in the destination directory. As distributed, Octave is installed in
        the following directories. In the table below, prefix defaults to
        /usr/local, version stands for the current version number
        of the interpreter, and arch is the type of computer on which
        Octave is installed (for example, i586-unknown-gnu).

        prefix/bin: Octave and other binaries that people will want to run directly. prefix/lib: Libraries like libcruft.a and liboctave.a. prefix/share: Architecture-independent data files. prefix/include/octave: Include files distributed with Octave. prefix/man/man1: Unix-style man pages describing Octave. prefix/info: Info files describing Octave. prefix/share/octave/version/m: Function files distributed with Octave. This includes the Octave version, so that multiple versions of Octave may be installed at the same time. prefix/lib/octave/version/exec/arch: Executables to be run by Octave rather than the user. prefix/lib/octave/version/oct/arch: Object files that will be dynamically loaded. prefix/share/octave/version/imagelib: Image files that are distributed with Octave.

- Installation Problems
